# Advanced Particle Size Analysis in High-Solid-Content Polymer Dispersions Using Photon Density Wave Spectroscopy

**DOI:** 10.3390/polym15153181

**Published:** 2023-07-26

**Authors:** Stephanie Schlappa, Lena Bressel, Oliver Reich, Marvin Münzberg

**Affiliations:** 1 Department of Physical Chemistry, innoFSPEC, University of Potsdam, Am Mühlenberg 3, 14476 Potsdam, Germanymarvin.muenzberg@uni-potsdam.de (M.M.); 2 Knowledge and Technology Transfer, Faculty of Science, University of Potsdam, Am Mühlenberg 3, 14476 Potsdam, Germany; oliver.reich@uni-potsdam.de

**Keywords:** emulsion polymerization, multiple light scattering, photon density wave spectroscopy, particle sizing, swelling of polymers

## Abstract

High-solid-content polystyrene and polyvinyl acetate dispersions of polymer particles with a 50 nm to 500 nm mean particle diameter and 12–55% (*w*/*w*) solid content have been produced via emulsion polymerization and characterized regarding their optical and physical properties. Both systems have been analyzed with common particle-size-measuring techniques like dynamic light scattering (DLS) and static light scattering (SLS) and compared to inline particle size distribution (PSD) measurements via photon density wave (PDW) spectroscopy in undiluted samples. It is shown that particle size measurements of undiluted polystyrene dispersions are in good agreement between analysis methods. However, for polyvinyl acetate particles, size determination is challenging due to bound water in the produced polymer. For the first time, water-swelling factors were determined via an iterative approach of PDW spectroscopy error (*Χ*^2^) minimization. It is shown that water-swollen particles can be analyzed in high-solid-content solutions and their physical properties can be assumed to determine the refractive index, density, and volume fraction in dispersion. It was found that assumed water swelling improved the reduced scattering coefficient fit by PDW spectroscopy by up to ten times and particle size determination was refined and enabled. Particle size analysis of the water-swollen particles agreed well with offline-based state-of-the-art techniques.

## 1. Introduction

Emulsion polymerization processes are one of the most common polymerization processes to produce high-solid-content polymer dispersions. In contrast to suspension polymerization, emulsion polymerization promotes higher solid contents, better heat transfer, faster polymerization rates, and better colloidal stability. A vast variety of polymers and stabilizers are already well-characterized. Accessible products range from low to high density polymers, branched and stretched, and homo- as well as co-polymers. Given the great variety of polymers, many different types of products and different product applications can be achieved [1,2].

The precise analysis of particle size and particle size distribution (PSD) is of the utmost importance for liquid polymer dispersions. These properties play an important role as they define the usage and applications of polymer dispersions, e.g., the fluidity and stability of high-solid-content polymer dispersions [3]. The mean particle size of polymers depends on many thermodynamic and kinetic factors during their synthesis, like the solubility of the monomer–polymer, composition of the reactants, temperature, solvent-to-medium ratio, or even reactor geometry [2,4]. The final mean particle size and PSD during emulsion polymerization are further influenced by the monomer amount introduced and the capability of the stabilizer in the dispersion to sustain the colloidal stability of the particles growing. The amount of stabilizer added accounts for the amount of particles formed and, therefore, also the final mean particle size [5].

To access the mean particle size in high-solid-content polymer dispersions and to determine the PSD, different techniques are known and promoted like turbidimetry, fiber-optic quasi-elastic light scattering or focused beam reflectance measurement, and particle vision and measurement technology. Online measurements in undiluted processes are particularly challenging due to the high turbidity, high number of particles, and multiple scattering [6,7,8,9,10,11]. Most commonly, offline PSD analysis is used. However, offline techniques require extensive sample preparation like dilution, are time consuming, and are not easy to handle. Additionally, dilution always induces a change in the particle system, whose effects on the measurement are not well-known. Analysis of nanoparticle size distribution is possible by multiple techniques. Laser light-scattering techniques are shown to deliver reliable results; however, adding to the extensive sample preparation and dilution, the results are influenced strongly and shifted by the fractions of bigger particles in the sample. For multimodal or very polydisperse samples, light-scattering techniques lack resolution [12,13,14,15].

Input parameters for measurements like the solvent viscosity, the refractive index, the sample concentration, and the sample temperature play a significant role and are compulsory. Necessary sample dilution evidently affects these factors and provokes falsified results. For dynamic light scattering (DLS) measurements, the dilution of the sample affects the autocorrelation function and the resulting particle size. For samples with high particle concentration, i.e., high solid contents, the Stokes–Einstein equation is no longer valid and diffusion coefficients yield wrong hydrodynamic radii [16,17,18,19]. Photon density wave (PDW) spectroscopy offers dispersion analysis of highly turbid particle systems or emulsions without dilution by the independent experimental determination of the optical coefficients, reduced scattering coefficient and absorption coefficient, respectively [20,21,22,23]. This technique is used in this study to determine the PSD in undiluted concentrated polymer dispersions and compared to the DLS, Static Light Scattering (SLS), and Electron Microscopy (EM) analysis of diluted samples.

**Figure 1:**
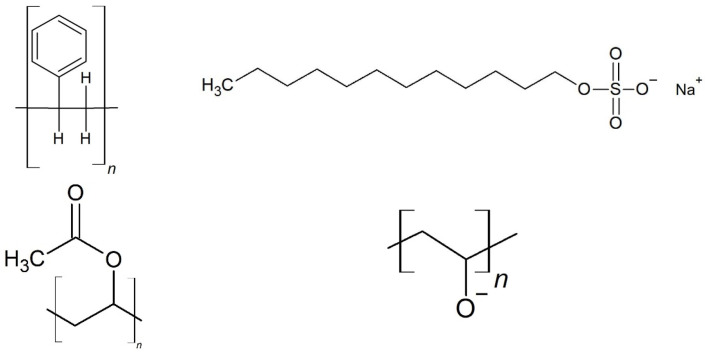
Representative chemical structures of polymers PS and PVAc, and stabilizers SDS and PVA used in this study. From top left to bottom right: structure of polymer PS, stabilizer SDS, polymer PVAc, and stabilizer PVA.

In this work, two polymer systems are produced and thoroughly analyzed—polystyrene (PS) particles, stabilized by sodium dodecyl sulfate (SDS), and polyvinyl acetate (PVAc), stabilized by poly (vinyl alcohol, PVA). The respective chemical structures of the polymers and stabilizers used in this study are shown in Figure 1. Stabilizers are shown in their deprotonated form. It is reported in literature that PS and SDS are not prone to incorporating water, due to their hydrophobic character, whereas PVAc and PVA, due to their significantly more hydrophilic character, especially PVA with plenty of accessible hydroxyl groups also shown in Figure 1, are not only known to bind water loosely by van der Waals interactions, but are also able to build hydrogen bonds, which bind the water strongly to the hydrophilic sites [24,25,26,27]. Due to the polarizability of SDS, it is possible that water molecules attach to the surfactants surrounding the polymer, but the amount and effect on the particle will be significantly lower than for the PVA stabilized particles. The hydrophobic aklylchains will attach to the polymer and few loose interactions happen with the surrounding media and water.

Three states of bound water were found to be present in PVA: free water, bound–freezable, and bound–non-freezable water, which has nearly the same characteristics as bulk water [28,29,30]. The amount of freezable water accounts only for a very small percentage and is, therefore, complicated to detect. Only a combination of different thermal analyses may be able to determine the amount of water in the PVAc-PVA particle [31,32,33]. It is assumed that the PVAc-PVA particles are swollen with water and also have a swollen emulsifier shell around the PVAc particle core. The degree of swelling is hard to determine due to the different kinds of water-binding mechanisms. The swelling of the polymer particle has a significant influence on the density and refractive index of the particle as well as the solid content and the volume ratio between the PVAc polymer core, polymer shell, and medium in the dispersion. These are the main factors that have a great influence on the PSD measurements. A lot of data on polymer swelling in literature concentrates on the swelling of dry polymer films in water or the swelling of hydrogels [34,35,36]. In this study, undiluted measurements of PVAc-PVA particles are analyzed regarding the swelling of the particle with the medium in high-solid-content dispersions.

For both systems, PS-SDS and PVAc-PVA, particles with various mean particle sizes and PSD can be obtained by emulsion polymerization [5,37,38]. The polymers can be produced in numerous shapes, co-monomer compositions, and sizes from a few nanometers to the upper micrometer size regime [5,39,40]. To produce mean particle sizes between 50 and 500 nm, seeded emulsion polymerization was performed. Seeded emulsion polymerization is a well-known method to produce monodisperse PS dispersions.

Starved-feed emulsion polymerization was used to produce PVAc-PVA particles between 100 and 500 nm mean particle size [20,41,42].

## 2. Materials and Methods

All chemicals were used and handled as described. Styrene was purchased from different suppliers throughout the experiments (104.15 g mol^−1^, ≥99.5%, Carl Roth, Karlsruhe, Germany and Merck, Darmstadt, Germany). Sodium dodecyl sulfate (SDS, 288.38 g mol^−1^, Carl Roth, Karlsruhe, Germany), di-sodiumtetraborate decahydrate (Na-Tetra Borate, 381.37 g mol^−1^, ≥99.5%, Carl Roth, Karlsruhe, Germany), sodium hydroxide (NaOH, 40.0 g mol^−1^, ≥99%, Carl Roth, Karlsruhe, Germany), aluminum hydroxide (AlOH_3_, dried gel, 78.01 g mol^−1^, 99%, abcr GmbH, Karlsruhe, Germany), and potassium peroxodisulfate (KPS, 270.32 g mol^−1^, ≥99%, Carl Roth, Karlsruhe, Germany) were used as purchased.

Prior to use, the styrene was destabilized by filtering over granulated AlOH_3_ in a flash column and, afterwards, washed repeatedly in 1 M NaOH using a separating funnel. The styrene showed a light transparent-yellowish color and was further handled at room temperature. Monomer vinyl acetate (VAc, 86.09 g mol^−1^, ≥99%, Sigma-Aldrich, Darmstadt, Germany) was purged with N_2_ (Nippon Gases, Duesseldorf, Germany) for 30 min prior to synthesis. Redox initiator pair ascorbic acid (AA, 176.12 g mol^−1^, 99%, Acros Organics, Geel, Belgium) and sodium persulfate (NaPS, 238.10 g mol^−1^, ≥99%, Carl Roth, Karlsruhe, Germany) and catalyst ammonium iron (III) sulfate hexahydrate (FAS, 392.14 g mol^−1^, 99+%, Acros Organics, Geel, Belgium) were used as purchased. Mowiol^®^ 4-88 (polyvinyl alcohol, approx. 31,000 g mol^−1^, Sigma-Aldrich, Darmstadt, Germany) was used as purchased.

### 2.1. Synthesis

Preparation of solutions was performed with analytical grade Milli-Q^®^ water from an in-house Milli-Q^®^ water dispenser (Milli-Q^®^, Integral 5, Merck Millipore, Darmstadt, Germany). An automated lab reactor (OptiMax 1001, Mettler Toledo, Gießen, Germany) at approx. 1 L reaction scale was used for synthesis. Produced polymer dispersions were stored in airtight containers and showed no sign of destabilization for over twelve months.

#### 2.1.1. PS Seed Synthesis

Batch emulsion polymerization was used to produce PS particles with a narrow size distribution to be used further as seed particles. The recipe for the seed synthesis is given in Table 1.

Emulsion polymerization was started by charging 500 g of Milli-Q^®^ water in the 1 L batch reactor. Under stirring at 100 rpm and N_2_ purging, the initial charge (iC) was heated to 100 °C for 60 min to degas. Temperature was reduced to 55 °C over 45 min. At 55 °C reaction temperature, 135 g styrene, 6.2 g SDS in water, and 0.125 g of Na-Tetra Borate were added. After a short waiting period for the emulsion to form, 30 mL of a 5% KPS solution were added automatically via a computer-controlled dosing unit at 6 mL min^−1^. Simultaneously, the stirrer was ramped to 200 rpm. The emulsion was left to polymerize at 55 °C reaction temperature for 24 h. After 24 h, a post-polymerization step at 83 °C for 3 h was initiated. The system was cooled to 20 °C over 30 min.

#### 2.1.2. PS-Seeded Emulsion Polymerization

To produce PS nanoparticles of different mean particle sizes, seeded emulsion polymerization was performed. A small amount of the PS seed produced after Table 1 was taken and the particles were grown to various mean particle sizes, as shown in Table 2.

#### 2.1.3. Emulsion Polymerization of Polyvinyl Acetate Dispersions

Recipe for the produced PVAc-PVA dispersions is shown in Table 3 with determined density of the pure polymer and gravimetrically derived solid content after at least 48 h drying at 74 °C in an oven. Details on the emulsion polymerization process can be found in Schlappa et al. [20].

### 2.2. Dispersion Analysis

After completion of a synthesis, offline sample analysis was performed. After approx. 400-fold dilution with Milli-Q^®^ water to visual transparency, three repetition measurements of each sample were performed by DLS analysis (Zetasizer Ultra, Malvern Panalytical, Worcestershire, UK), at measurement angle of 173°, in disposable 4 mL PS cuvettes, at 25 °C measurement temperature. Samples for SLS measurements (LS13320, Beckman Coulter, Brea, CA, USA) were manually diluted approx. 1:125 with Milli-Q^®^ water and charged to the SLS sample chamber until a polarization intensity differential scattering (PIDS) signal of approx. 40% was achieved. Obscuration values lower than 2% were retained. Results shown for PSDs obtained by light-scattering techniques are mean values of three measurements. Out of the three individual measurements, the mean value is plotted with one standard deviation as error bars. For electron microscopy (EM) analysis, samples were diluted approx. 1000-fold with Milli-Q^®^ water and a drop of sample was placed onto a copper-coal mesh (Plano GmbH, Wetzlar, Germany). The sample was left to dry for an hour at ambient conditions. Quanta FEI 250 electron microscope was used for analysis in STEM mode.

### 2.3. PDW Spectroscopy Dispersion Analysis

Photon density wave (PDW) spectroscopy is among few techniques which offers dispersion analysis by the determination of the optical coefficients of a highly turbid particle system without dilution even at high solid contents of the dispersed species. The PDW spectroscopy device used here is self-built (University of Potsdam—innoFSPEC, Potsdam, Germany). No sample preparation is needed as the optical fibers can directly be inserted into the dispersion for measuring. Two optical fibers are immersed into the undiluted dispersion. These fibers act as light emission and detection source for intensity-modulated laser light. The detection fiber is mounted to a precision translations stage which moves in scalable distances to and from the emission fiber. Fibers used in this set-up have a 600 µm core diameter. Intensity-modulated laser light (10–1210 MHz) is guided into the sample via the emission fiber and interacts with the sample regarding the samples’ absorption and scattering properties. Due to the multiple scattered light, a PDW is expressed. A small portion of the PDW light is guided to an avalanche photo diode by the detection fiber. The electronic signal is amplified and then analyzed by a network analyzer with respect to phase shift and amplitude. The change in amplitude and phase in dependency of the distance between the emission and detection fiber and the modulation frequency can be related to the absorption coefficient *µ*_a_ and reduced scattering coefficient *µ*_s_’ of the sample. These optical coefficients can be obtained for different wavelengths one after another.

The reduced scattering coefficients are used to determine the mean particle size and particle size distribution, with the help of the Mie theory and the theory of dependent scattering [22,43]. Highly turbid polymer samples have already been successfully analyzed and characterized by PDW spectroscopy regarding their mean particle diameter and PSD [44,45,46,47]. Inline monitoring of emulsion polymerization processes has also been successfully reported [20,21,48,49,50].

In this study, two polymer systems were evaluated, one which is not prone to incorporating water and one where water swelling of the particles is common, to show the differences in analyzing the particle size by PDW spectroscopy if water swelling of particles occurs. To determine the mean particle size and PSD, theoretical *µ*_s_’ values are calculated that match the experimentally determined *µ*_s_’ values by the applied PDW spectroscopy algorithm. If experimental and theoretical values agree well with each other, it can be assumed that the swelling, and particle characteristics represent the actual situation in the dispersion. To theoretically reproduce the experimentally determined *µ*_s_’, the refractive index, and density of the polymer and medium as it is present in the dispersion, as well as the solid content, an assumption of the mean particle diameter and width of a normal logarithmic Gauss distribution are necessary for a data fit. In a first step, the solid content will be determined gravimetrically. A small weighted sample is placed in a drying cabinet at low temperature (74 °C) in order to keep the polymer intact. Drying was achieved over a period of time >72 h to evaporate the medium and bound water inside the polymer. The dry weight is measured at mass consistency, and the solid content calculated.

A concentration series of 30% (*w*/*w*), 20% (*w*/*w*), 10% (*w*/*w*), 1% (*w*/*w*), and 0.1% (*w*/*w*) of each polymer dispersion was used to determine the density of the polymer particles. Each sample was measured at 20 °C with a densitometer (DM45 Delta Rage, Mettler Toledo, Gießen, Germany). Density analysis of the particles for the pure polymer (100% (*w*/*w*)) was performed by [49]:

(1) $$\rho_{Disp}=\frac{\rho_{D}\rho_{P}}{\omega \left[\rho_{D}-\rho_{P}\right]+\rho_{P}}↔\frac{1}{\rho_{Disp}}=\omega \left[\frac{1}{\rho_{P}}-\frac{1}{\rho_{D}}\right]+\frac{1}{\rho_{D}}$$

with *ρ*_Disp_, *ρ*_D_, and *ρ*_P_ as densities of the dispersion, the dispersant, and the polymer and the solid content *w* of the dispersion. The density of pure water was considered for zero polymer content (0% (*w*/*w*)). The density of the polymer was obtained from the slope of a linear fit; the error was obtained via error propagation.

The same samples were also used for refractive index measurements with a multi-wavelength refractometer (DRS-λ, Schmidt + Haensch, Berlin, Germany) at 20 °C at seven different wavelengths between 403 and 938 nm. The measured refractive indices are extrapolated to the refractive index of the particles using the Newton equation and, afterwards, inter- or extrapolated to the wavelengths of PDW spectroscopy by a Cauchy formula [49,51].

To speed up the PDW calculations, estimates of the mean particle size and PSD can be introduced based on offline reference methods or theoretical particle size considerations. With these parameters, a first PDW spectroscopy fit is obtained within a few minutes.

In the case that this first fit of theoretical *µ*_s_’ does not match the experimental results, high error values *Χ*^2^ for the *µ*_s_’ fit are given by the software. The value of *Χ*^2^ expresses how well the theoretically calculated values agree with the experimentally determined values and is a measurement of the fit quality. If *Χ*^2^ values are high, modifications of the input parameters are necessary, as the particle in solution expresses different properties as determined by this first rough estimation of its physical properties. Literature data already suggest that the bound water inside the PVAc-PVA polymer cannot be dried off completely by only drying in an oven. A combination of different thermogravimetric techniques is necessary to determine the exact water content, which is very time consuming, costly, and complicated. The water-swollen polymer in solution does not express the same properties like in a dried state. To validate the mean particle diameter and PSD measurement by PDW spectroscopy, iterative steps of water swelling *θ*_swollen_ are assumed, affecting the density $\rho_{P,\;swollen}$, refractive index $n_{P,swollen}$, and volume fraction $\varphi_{P,\;swollen}$ of the polymer to a great extent. Various percentages of water swelling *θ*_swollen_ in 0.05 steps (from 0% to 50%) are assumed and added to the experimentally derived solid content. A new volume fraction for the particle affected by water swelling is calculated:

(2) $$\varphi_{P,swollen}=(1+\theta_{swollen})\;\frac{\left(\frac{\omega_{gravimetrical}}{\rho_{Polymer}}\right)}{\left(\frac{\omega_{gravimetrical}}{\rho_{experimental}}+\frac{\left(1-\omega_{gravimetrical}\right)}{\rho_{D}}\right)}$$

with this new volume fraction of the swollen polymer $\varphi_{P,\;swollen}$, adjusted values for the density (Formula (1)) and refractive index (Formula (3)) are recalculated.

(3) $$n_{P,swollen}=\sqrt{\varphi_{P,\;swollen}*n_{P}{}^{2}+\left[1-\varphi_{P,\;swollen}\right]*n_{D}{}^{2}}$$

with the recalculated refractive index of water-swollen polymer as *n*_P,swollen_, the increased volume fraction *φ*_P,swollen_, and the refractive index of the non-swollen particle *n*_P_ and dispersant *n*_D_. These recalculated values are used with the assumed mean particle size and PSD to recalculate *µ*_s_’ by the automated PDW spectroscopy algorithm. The resulting *µ*_s_’ values are compared to the experimental values again and the *X*^2^ error parameter for the fit of theoretical-to-experimental *µ*_s_’ is analyzed. The smaller the *X*^2^, the better the agreement between experimental and theoretical values, meaning the better the fit quality, and the water-swelling factor *θ*_swollen_ is assumed to represent the real properties of the particles in the analyzed dispersion.

This iterative approach is carried out for several percentages of water swelling until the lowest possible *X*^2^ value is found, starting with 10% water-swelling steps, finding the lowest *X*^2^ and minimizing the steps up to 1%. A parabola fit is used to find the minimum of *X*^2^ of the theoretically determined PDW spectroscopy fits. This percentage of water swelling represents the experimental data best and it can be assumed that the polymer is swollen with water by the derived percentage.

For comparison between size distribution data from DLS, SLS, and PDW spectroscopy, the mean particle diameter calculated from the volume frequency distributions are normalized regarding the bin width. PSDs are usually shown in different types of distributions. Most common types include intensity, number, and volume-based PSDs. PDW spectroscopy provides a volume-based PSD with a mean particle diameter comparable to the so-called De Brouckére mean (*d*_43_). The DLS measurement used for comparison gives a PSD in volume from measuring the hydrodynamic radius, calculated from an intensity weighted measurement [52,53,54]. To compare particle size distributions derived from PDW spectroscopy to distributions derived from offline, dilution-based techniques, an area normalization of the distribution is done. Normalization results in volume frequency distributions per nanometer (*f*_3_) for every measurement method.

## 3. Results and Discussion

All high-solid-content samples were synthesized as explained in the experimental section. For all dispersions, a white, turbid liquid without visible coagulation was obtained and stored in airtight containers until further use. Two polymer systems have been examined: the first, PS-SDS, is not known to incorporate water and no swelling of the particles is expected. Results are first shown for PS-SDS dispersions regarding reproducibility, PDW spectroscopy analysis of optical coefficients *µ_a_* and *µ_s_*’, and PSD analysis by PDW spectroscopy compared to offline standard PSD measurement methods as a best practice. The second particle system, PVAc-PVA, is analyzed in the same way regarding reproducibility and PDW spectroscopy with a focus on the determination of the particle swelling and size determination of the polymer particles.

### 3.1. Polystyrene—SDS Dispersions

#### 3.1.1. Reproducibility of Polystyrene Dispersions

**Figure 2:**
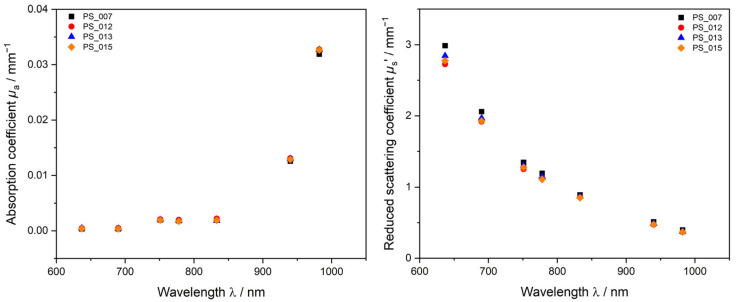
Four repeated emulsion polymerization PS-SDS experiments. Dispersions produced after the same recipe and procedure. Optical parameters *µ*_a_ (**left**) and *µ*_s_’ (**right**), determined in the undiluted sample at approx. 36.3% (*w*/*w*) PS-SDS with PDW spectroscopy.

To investigate the reproducibility during this work, up to four polymerizations have been performed at equal conditions. Figure 2 shows the absorption coefficient *µ*_a_ and reduced scattering coefficient *µ*_s_’ obtained by PDW spectroscopy measurements of four undiluted PS dispersions obtained by identical syntheses. Results show that the dispersions are alike in their physical and chemical properties. The solid content of the four experiments was determined gravimetrically and statistically analyzed to be *w* = 36.3 ± 0.3% (*w*/*w*) (*n* = 4). For *µ*_a_, very good agreement between the four experiments is obtained over the whole measurement range, whereas, for *µ*_s_’, measurements at the lower wavelengths show small deviations in between the experiments. At wavelengths >700 nm the obtained data points agree very well with each other. The small deviations occurring at the lower wavelength range might occur due to possible fluorescent interferences of the laser and the sample. Another factor which might cause these small deviations in *µ*_s_’ are the polymer content and small deviations in size of the samples. The difference in PS content in between the measurements, however, is very small; the highest *w* was gravimetrically determined to be *w* = 36.8% (*w*/*w*) for PS_015 and the lowest *w* of 36.5% (*w*/*w*) for PS_007. Even such small differences in the solid content, and possibly size of a few nanometers, can be detected by PDW spectroscopy via *µ*_s_’. Due to the higher polymer content of the dispersions, an increase in particle scattering can be observed with PDW spectroscopy, where other methods like gravimetric solid content determination fail to detect this minimal difference in solid content. PS-SDS syntheses are highly reproducible with very small differences in mean particle size.

#### 3.1.2. PDW Spectroscopy Analysis of Undiluted PS-SDS Dispersions

**Figure 3:**
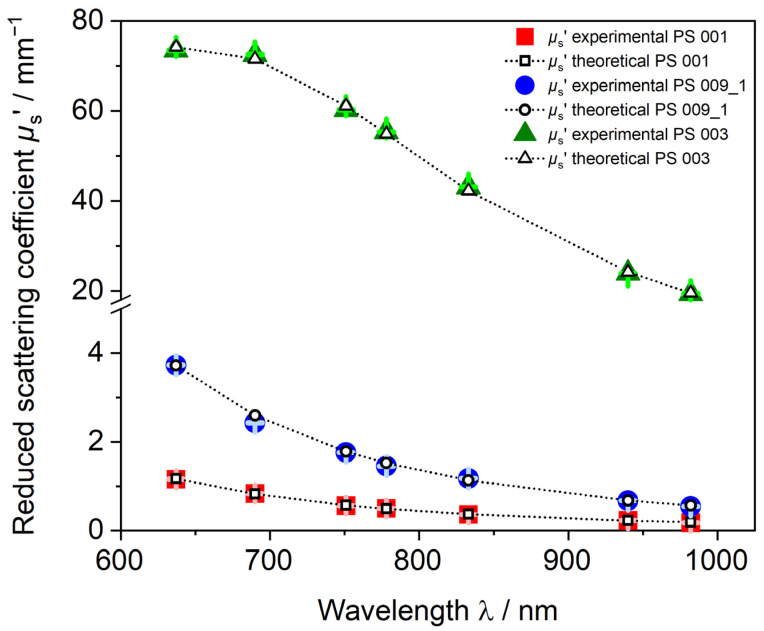
Calculated theoretical *µ*_s_’ values obtained with Mie-theory- and global-analysis-based PDW spectroscopy fit (open symbols) fitted to experimental *µ_s_*’ (closed symbols) with calculated error bars highlighted in light green, light blue and light red. PS-SDS dispersion with mean particle diameter of PS 001 = 49.0 nm (red squares), PS 009_01 = 99.1 nm (blue circles), and PS 003 = 271.7 nm (green triangles).

The scattering behavior of a sample contains a lot of information about the particles in the dispersion. PDW spectroscopy measures the reduced scattering coefficient *µ*_s_’ dependent on the laser wavelength *λ* in undiluted polymer samples. Figure 3 shows experimentally measured *µ*_s_’ values of three different PS-SDS dispersions and theoretical values obtained by PDW spectroscopy size analysis from a fit to the experimental data points after assuming the respective input parameters density, refractive index, size, and width of the distribution. For PS-SDS dispersions in the lower nanometer size regime, i.e., <100 nm mean particle diameter (PS 001 and PS 009_1), *µ*_s_’ values < 4 mm^−1^ were obtained with *µ*_s_’ decreasing steadily with increasing wavelength. For a dispersion with a mean particle diameter of approximately 270 nm (PS 003). values for *µ*_s_’ were up to twenty times higher and a significant change of the trend form and an elevation in the curve can be observed at lower wavelengths around 700 nm. Even with the elevation and changed curve form, the measured *µ*_s_’ values can be reproduced very well with assumptions of the physical parameters without necessary adjustment.

PDW spectroscopy for polystyrene dispersions is applicable for a wide range of dispersions, mean particle sizes, PSDs, and values of *µ*_s_’ between 0.1 and 80 mm^−1^ at least. The theoretical *µ*_s_’ values by PDW spectroscopy shown in Figure 3 as open symbols are calculated with gravimetrically derived solid content of PS 001 = 12.47% (*w*/*w*), PS 009_01 = 37.80% (*w*/*w*), and PS 003 = 29.89% (*w*/*w*). Density *ρ* and refractive index *n* of the pure PS-SDS sample without any further assumption of water swelling were calculated to *ρ*_PS-SDS_ = 1.055 g cm^−3^ and *n* ranging from 1.59 at 637 nm to 1.57 at 982 nm. The very good agreement of the fitted *µ*_s_’ values to the experimental values verifies that SDS as a stabilizer does not incorporate water and PS-SDS particles are not swollen in water-based dispersions. The calculated values for *w*_gravimetrical_, *n*_Polymer_, and *ρ*_Polymer_ represent the actual particle properties as no water swelling occurs, causing these parameters to change. Therefore, the theoretical *µ*_s_’ values fit well to the experimental values and low *X*^2^ values are obtained. *X*^2^ values are PS 001 = 336, PS 003 = 271, and PS-009_01 = 941.

#### 3.1.3. Method Comparison DLS, SLS, and PDW Spectroscopy

The dependency of *µ*_s_’ on the particle diameter can be described by the Mie theory and dependent scattering and is used to derive a PSD from PDW spectroscopy measurements. To compare the distributions of the three different particle-sizing techniques, normalization regarding the size bin width has been applied and volume-based frequency distributions *f*^3^ are calculated. Mean particle diameters determined by each technique are compared in Table A1 in the Appendix A.

**Figure 4:**
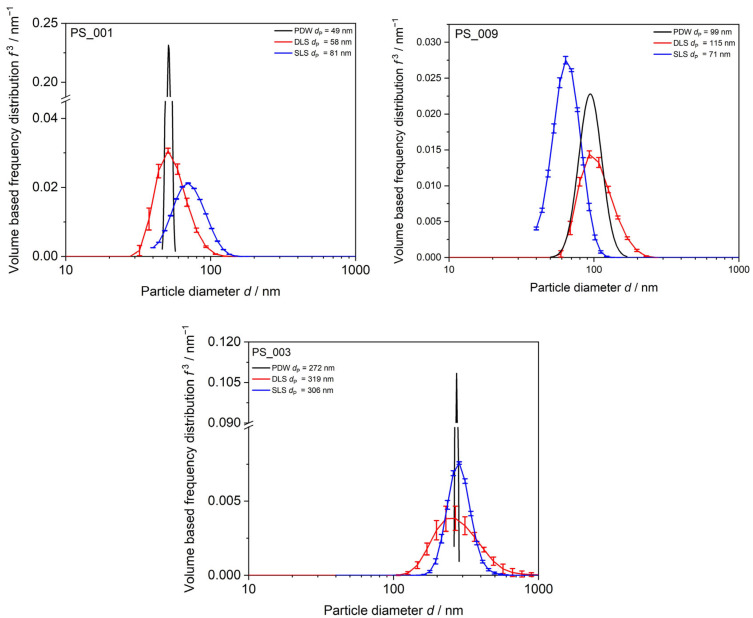
PSD of PS-SDS emulsions with mean particle diameters from approx. 49 nm to 272 nm. Comparison between dilution-based DLS, SLS, and undiluted PDW spectroscopy. DLS and SLS values are shown as mean out of three measurements with calculated mean value and standard deviation as error bars.

DLS always gives mean particle diameter values of approx. 15 to 20% bigger than PDW (Table A1 in the Appendix A). This is caused by the fact that DLS measurements determine the hydrodynamic radius of the particle, whereas PDW spectroscopy determines the size of a hard-sphere particle. The SLS determination of mean particle size shows deviations to DLS and PDW spectroscopy and determined values might be bigger or smaller than for the other techniques. The biggest deviations of up to 40% are found between PDW spectroscopy and SLS for mean particle sizes around 100 nm and smaller. SLS sizing is not suitable for such small particle diameters, as the measurement particle size range starts only at 40 nm on the device used and deviations are unavoidable which falsify the results. For the PSD determination by all three techniques, monomodality is assumed and verified in Figure 4. Comparison between dilution-based light-scattering techniques DLS and SLS and undiluted measurements via PDW spectroscopy are shown in Figure 4. The dilution-based methods result in far wider PSDs than data obtained from PDW spectroscopy, which gives the narrowest PSDs for all three dispersions shown. DLS measurements show tendencies to bigger particle sizes leading to bigger mean *d*_P_ than PDW spectroscopy measurements. Results found in earlier studies revealed similar results regarding DLS [20,21,48]. SLS measurements show no clear trend. For the smallest particles, SLS shows a big deviation from the other methods with a mean *d*_P_ of 81 nm rather than 50 nm like DLS and PDW spectroscopy. The SLS device used in this study starts measuring at 40 nm, leading to the assumption that particles of 50 nm mean *d*_P_ lie within the lowest possible measurement range. For the dispersions with bigger mean particle sizes, SLS shows better agreement to the other methods.

### 3.2. Polyvinyl Acetate—Polyvinyl Alcohol Dispersions

#### 3.2.1. Reproducibility PVAc-PVA

**Figure 5:**
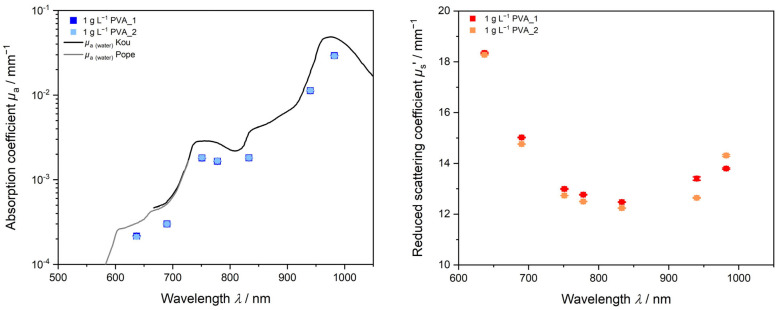
Reproducibility of PVAc-PVA dispersion with 1 g L^−1^ PVA in the initial charge. Repetition experiments were performed at equal reaction conditions.

Figure 5 shows two emulsion polymerization products of PVAc-PVA with 1 g L^−1^ PVA in the initial charge produced under equal conditions. The left graph shows that, regarding the absorption properties, no differentiation between the two samples is possible. The trend of *µ*_a_ is following the form of pure water absorption, due to the high amount of water as the dispersion medium and little expected absorption from the polymer indicated by literature values from Kou and Pope [55,56]. Lower values are obtained as the polymer is present by approx. 40% (*w*/*w*) solid content. In Figure 5 (right), the *µ*_s_’ trend versus wavelength shows small deviations in between the measurements. In the lower wavelength range, the first experiment 1 g L^−1^ PVA_1 gives a slightly higher value for *µ*_s_’. For wavelengths >950 nm, this order is inverted, and the second experiment gives higher values for *µ*_s_’. The solid content for both syntheses is nearly identical with 1 g L^−1^_PVA_1 = 41.61% (*w*/*w*) and 1 g L^−1^_PVA_2 = 40.87% (*w*/*w*), respectively. The slightly higher solid content might be responsible for the higher *µ*_s_’ values for 1 g L^−1^_PVA_1.

#### 3.2.2. PDW Spectroscopy Size Analysis of Undiluted PVAc-PVA Dispersions

**Figure 6:**
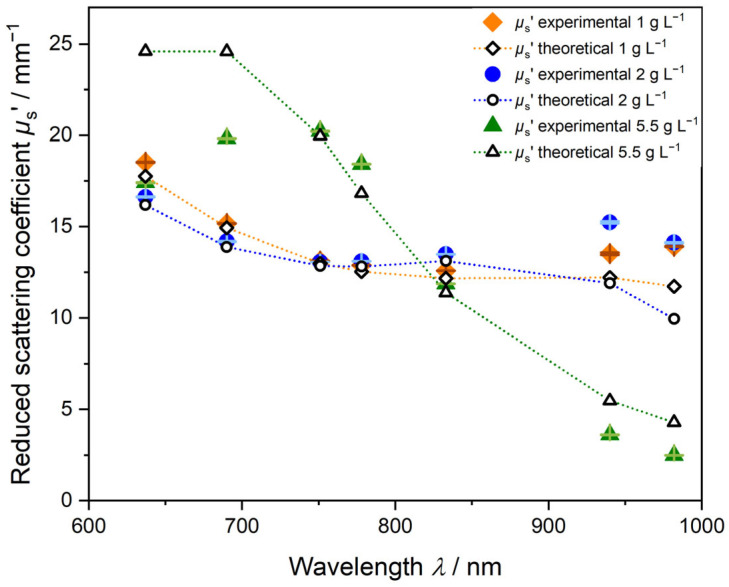
Theoretical *µ*_s_’ values (open symbols) fitted to experimental *µ*_s_’ values (closed symbols) from a Mie-theory- and global-analysis-based PDW spectroscopy measurement without assumption of water swelling. Three different PVAc-PVA dispersions with increasing content of PVA in the iC are shown. The orange curve shows *µ*_s_’ for 1 g L^−1^ PVA in the iC, blue 2 g L^−1^, and green 5.5 g L^−1^. Error bars are indicated in brown, light blue and light green respectively.

For PVAc-PVA syntheses, particles with mean diameters between approximately 270 nm and 510 nm were obtained by starved-feed emulsion polymerization. Regarding the analysis of the undiluted dispersions using PDW spectroscopy, it was found that, for the PVAc-PVA dispersions, the trend form of *µ*_s_’ vs. wavelength deviates from the decreasing trends shown for the PS-SDS systems in Figure 3. Figure 6 shows experimental and theoretical data for *µ*_s_’ without any assumption of water swelling. Density and refractive index were calculated from gravimetrically derived solid contents *w* of PVAc-PVA_1 g L^−1^ = 41.61% (*w*/*w*), PVAc-PVA_2 g L^−1^ = 40.92% (*w*/*w*), and PVAc-PVA 5.5 g L^−1^ = 41.57% (*w*/*w*). Theoretical *µ*_s_’ as a function of wavelength is shown as open symbols with dotted lines as a guide for the eye.

The experimental values (closed symbols in Figure 6) cannot be fitted exactly, if only the pure polymer properties and no swelling are considered and used for analysis (open symbols). Contrary to the SDS-stabilized PS samples, the PDW spectroscopy fit for the PVA-stabilized PVAc samples is not easily applied, and the calculated data points do not agree well with the experimental *µ*_s_’ values over the wavelength range. This relates to the physical properties and scattering behavior of the PVAc-PVA particles. The deviation is caused by bound water in the PVAc-PVA particle. Due to the difference in their macromolecular chemical structure of the polymers and stabilizers, the particles express different properties and different water-binding abilities. For the hydrophilic PVAc-PVA particles, water is penetrating inside the particle and causes swelling. For PDW spectroscopy analysis, the volume fraction, refractive index, and density of the particle influence the resulting theoretical *µ*_s_’ calculation, which is used for the exact size determination to a great extent. Water swelling of the PVAc-PVA particle causes changes to these factors and the calculated *µ*_s_’ values are erroneous.

**Figure 7:**
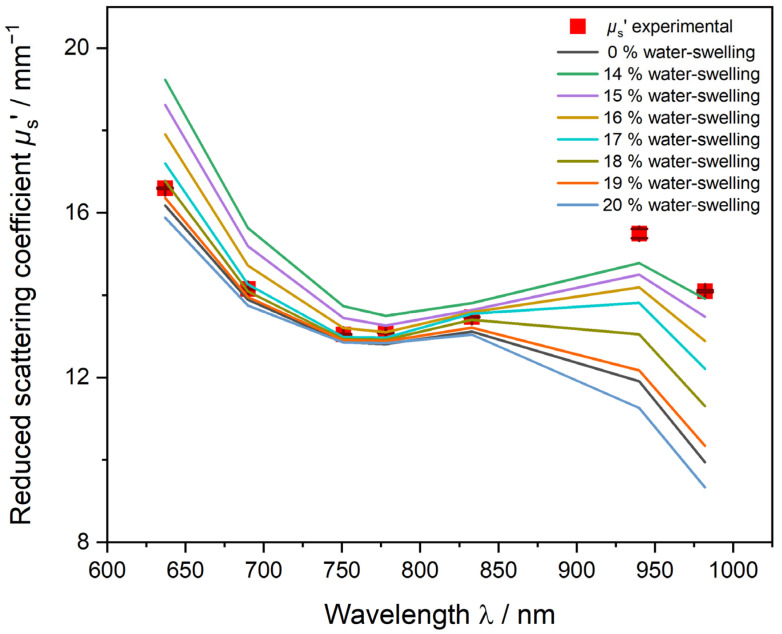
Iterative approach of PDW spectroscopy fit of the reduced scattering coefficient *µ*_s_’ to minimize *Χ*^2^ by increasing the water-swelling factor of the PVAc-PVA particle. Sample with 2 g L^−1^ PVA in the iC. Black line shows the *µ*_s_’ fit without any water swelling assumed.

**Figure 8:**
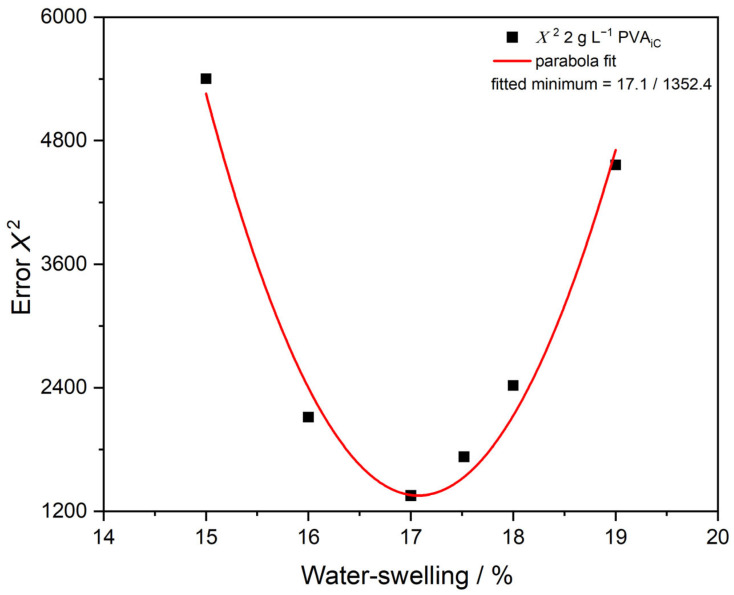
Assumed water swelling, with corresponding *Χ*^2^ values fitted with a parabola fit to find the lowest *Χ*^2^ value and, therefore, assumed best PDW spectroscopy fit. Parabola fit used gives *R*^2^ = 0.96.

Figure 7 and Figure 8 show the results of the iterative approach explained earlier in the section “2.3 PDW Spectroscopy Dispersion Analysis” to determine the water swelling of the particles by a PDW spectroscopy fit analysis of theoretically calculated *µ*_s_’ to experimentally determined values. For the dispersion PVAc-PVA 2 g L^−1^ shown in Figure 7, *µ*_s_’ shows a minimum at approx. 750 nm and increases again to a maximum at 940 nm. The fit of the non-swollen curve (black line) cannot match the experimental results (red squares) and gives a very high value of *Χ*^2^ = 5486. Assumed percentages of water swelling leads to a better fit and, correspondingly, smaller *Χ*^2^ values (Figure 8). The smallest *Χ*^2^ = 1352 for this sample was calculated at 17.01% water swelling and a mean size of 391 ± 43 nm, improving the fit quality by factor four.

Figure 8 shows the *Χ*^2^ values of assumed iterative water swelling between 15% and 19%. The minimum is calculated with a parabola fit to be at 17.1% water swelling.

Table 4 gives the data of assumed water-swelling and *Χ*^2^ values on the other PVAc-PVA syntheses and clearly shows that, for all PVAc-PVA samples, assumed water-swelling leads to a significant increase in the fit quality and reduction in *Χ*^2^. Improvement leads from *Χ*^2^ reduction by a factor of three to an improvement of over ten times for two samples. The results show that, for all these syntheses, a certain degree of water swelling needs to be considered and it is very likely that the particles are swollen in the dispersion.

#### 3.2.3. Method Comparison DLS, SLS, EM, and PDW Spectroscopy

Mean particle diameter for all dispersions was also determined by DLS, SLS, and PDW spectroscopy. The determined mean particle diameters for each sample with the determined swelling factor by iterative approach described before to minimize *Χ*^2^ are given in Table 5. Deviations between methods again range from 5% to 27% whereas DLS always gives an approx. 5 to 15% bigger mean *d*_P_ value due to measuring the hydrodynamic radius rather that the hard-sphere particle diameter.

**Figure 9:**
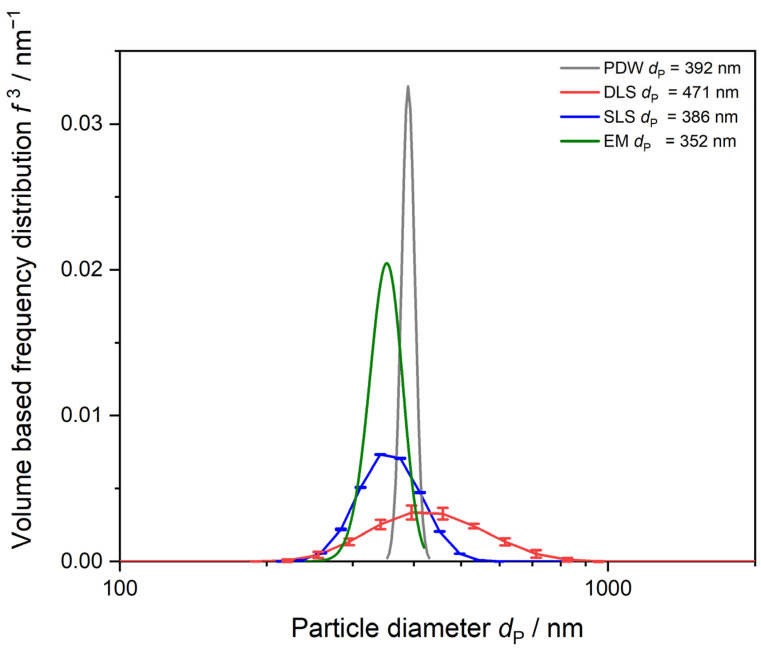
PSD of PVAc-PVA 2 g L^−1^ dispersion. Comparison between SLS, DLS, EM, and PDW spectroscopy. PDW spectroscopy distribution is determined with the calculated water-swelling factor of 17.01%. DLS and SLS values are shown as mean out of three measurements with calculated mean value and standard deviation as error bars.

The dispersion was analyzed additionally by EM, using the Hough Circle transformation method with a circularity cut off at c ≤ 0.8. A total of 70 particles were analyzed by determining a histogram and normalized Gaussian distribution. Mean *d*_P_ of 352 ± 27 nm was determined. Figure 9 shows that all the methods are in good agreement. EM analysis delivers the smallest mean *d*_P_ as the sample is measured in a dried state and the water-swollen particle might collapse during sample preparation and measurement under vacuum conditions. After assumption of the swelling factor of 17.01%, PDW spectroscopy is able to deliver a result for the mean *d*_P_ = 392 nm, with a narrow PSD. If one assumes that EM measures only the particle core and the swollen particle is collapsed completely, the mean *d*_P_ values between EM and PDW spectroscopy reflect the calculated swelling factor within the range of error. According to PDW spectroscopy, 17.01% of the volume of the 392 nm polymer are caused by water swelling, leading to a mean *d*_P_ of the collapsed particle of 325 nm which lies just within the range of errors of the calculated mean *d*_P_ from electron microscopy of 352 ± 27 nm.

## 4. Conclusions

The presented PDW spectroscopy technique offers a wide range of applications, even for material with extreme scattering properties and high solid contents. This study shows that, for both polymer particles analyzed, size determination is possible at industrially relevant reaction conditions without dilution or necessary changes to the sample.

For basic systems like polystyrene dispersions with an SDS stabilizer, which do not incorporate water, the fitting of the experimental data with PDW spectroscopy leads to very good results with low error values of *Χ*^2^. In polyvinyl acetate dispersions, however, the theoretical fit to the experimental data is lacking accuracy and the fitted values deviate from the experimental data points, if no water swelling is assumed. Comparing the two polymer systems reveals that analysis of the PS-SDS system compared to the PVAc-PVA system is less challenging and analysis of PS-SDS shows better agreement to offline-based techniques without further adjustment. In this study, we showed that PDW spectroscopy is a solid technique to determine the swelling of the polymer by iterative assumption of the swelling factor and consecutive adaptation of physical parameters.

## 5. Outlook

For the PVAc-PVA system, the applied model for the *µ*_s_’ fit needs to be modified, because of the incorporation of water in the PVAc-PVA particle. The incorporated water causes inconsistencies and deviations from the model assumptions for the physical particle properties like solid content and volume fraction, respectively, density, and refractive index, which are necessary for PSD analysis by PDW spectroscopy. The amount of free water and bound water is difficult to determine separately; differential scanning calorimetry (DSC) and further thermogravimetric analysis methods combined are necessary to investigate the amount of water in PVAc-PVA particles. Literature suggests a combination of thermal analysis methods like DSC, TGA, and IR analysis in order to determine the content of bound water in the particle structure.

In future work, the proposed iterative swelling approach and simultaneous adaptation of PDW spectroscopy input parameters needs to be incorporated in the particle size analysis of water-swollen polymers in the automated PDW spectroscopy size calculation algorithm.

## Figures and Tables

**Table 1:** Recipe for batch emulsion polymerization of PS seed particles.

	Chemical	Amount [g]
Medium	Milli-Q^®^ water	530
Monomer	Styrene	135
Stabilizer	SDS	6.2 ^a^
Buffer	Na-Tetra Borate	0.125
Initiator	Potassium peroxodisulfate	5.0 ^b^

^a^ dissolved in 30 g Milli Q^®^ water. ^b^ dissolved in 95 g Milli Q^®^ water.

**Table 2:** Recipe for seeded emulsion polymerization of PS particles.

	Chemical	Amount [g]	Feedrate [mL min^−1^]
Medium	Milli-Q^®^ water	308.6 ^a^	
Seed	PS Seed	31.5 ^b^	
Monomer	Styrene	337 ^c^	2.1
Stabilizer	10% SDS solution ^d^	164	
Buffer	Na-Tetra Borate	0.25	
Initiator	5% potassium peroxodisulfate solution	100	

^a^ except for syntheses PS003, PS004, and PS005 where 301 g water, PS009_1, and 009_2 where 130 g water, and PS020 where 500 g water were charged. ^b^ except for syntheses PS009_1 and PS009_2 where 100 g of PS seed were used. ^c^ except for syntheses PS009_1 and PS009_2 where 240 g styrene were fed at 1.5 mL min^−1^. ^d^ except for syntheses PS014 and PS016 where 3%, and PS018 and PS019 where 5% SDS solution were used.

**Table 3:** Recipe for PVAc-PVA dispersions. Experimental series of five syntheses with increasing amount of stabilizer in the iC.

Sample ID	PVA_iC_ [g]	Milli-Q^®^_iC_ [g]	*c* (PVA_iC_) [g L^−1^]	Solid Content ω [%]	Density ρ_P_ [g cm^−3^]
PVAc-PVA 0 g L^−1^	0	302	0	41.07	1.216
PVAc-PVA 1 g L^−1^	0.299	302	0.99	41.61	1.214
PVAc-PVA 2 g L^−1^	0.599	302	1.98	40.92	1.216
PVAc-PVA 5.5 g L^−1^	1.66	302	5.50	41.57	1.212
PVAc-PVA 11 g L^−1^	3.31	302	10.96	40.77	1.214

**Table 4:** Degree of water swelling determined by PDW spectroscopy iterative fitting to lowest *Χ*^2^ and corresponding improvement factor to non-swollen fit.

Sample	Degree of Swelling *θ*_swollen_/%	*Χ* ^2^	*Χ*^2^ Improvement Factor
PVAc-PVA 0 g L^−1^	19.29	87	13.7
PVAc-PVA 1 g L^−1^	22.00	250	7.2
PVAc-PVA 2 g L^−1^	17.07	1352	4.06
PVAc-PVA 5.5 g L^−1^	12.38	2,5944	3.38
PVAc-PVA 11 g L^−1^	38.41	4084	12.1

**Table 5:** Mean *d*_P_ values of PVAc-PVA particles in dispersion given from volume frequency distribution of PDW spectroscopy, SLS, and DLS measurements.

Sample ID	Mean *d*_P_ PDW	Mean *d*_P_ SLS	Mean *d*_P_ DLS	PDW/SLS	PDW/DLS
PVAc-PVA 0 g L^−1^	507.91	438.51	579.78	1.16	0.88
PVAc-PVA 1 g L^−1^_1 *	412.34	401.17	483.30	1.03	0.85
PVAc-PVA 1 g L^−1^_2 *	409.86	403.54	493.21	1.02	0.83
PVAc-PVA 2 g L^−1^	391.57	385.50	470.77	1.02	0.83
PVAc-PVA 5.5 g L^−1^ *	306.82	324.11	359.60	0.95	0.85
PVAc-PVA 11 g L^−1^	271.37	213.27	284.80	1.27	0.95

* syntheses compared in Figure 5.

## Data Availability

The data that support the findings of this study are available on request from the corresponding author.

## Appendix A

**Table A1:** Mean particle diameters *d*_P_ determined by particle-sizing techniques for different PS-SDS dispersions produced.

Sample ID	Mean *d*_P_ PDW	Mean *d*_P_ SLS	Mean *d*_P_ DLS	PDW/SLS	PDW/DLS
PS_001 *	49.00	80.85	58.50	0.61	0.84
PS_003	271.96	287.79	319.57	0.94	0.85
PS_004	296.42	318.80	356.29	0.93	0.83
PS_007 **	100.02	72.23	114.77	1.38	0.87
PS_009_1	99.14	71.83	115.27	1.38	0.86
PS_009_2	101.17	72.10	131.54	1.40	0.77
PS_011 *	75.87	82.99	78.06	0.91	0.97
PS_012 **	97.32	71.07	109.13	1.37	0.89
PS_013 **	99.00	71.65	116.47	1.38	0.85
PS_014	251.77	250.56	254.23	1.00	0.99
PS_015 **	96.46	72.09	113.04	1.34	0.85
PS_016	274.34	321.80	283.50	0.85	0.97
PS_018	259.82	288.54	278.27	0.90	0.93
PS_019	259.48	315.30	271.33	0.82	0.96
PS_020 *	56.96	69.27	80.46	0.82	0.71

* seed synthesis after Table 1. ** syntheses compared in Figure 2.
